# Genetic correction of Werner syndrome gene reveals impaired pro‐angiogenic function and HGF insufficiency in mesenchymal stem cells

**DOI:** 10.1111/acel.13116

**Published:** 2020-04-22

**Authors:** Jiajie Tu, Chao Wan, Fengjie Zhang, Lianbao Cao, Patrick Wai Nok Law, Yuyao Tian, Gang Lu, Owen M. Rennert, Wai‐Yee Chan, Hoi‐Hung Cheung

**Affiliations:** ^1^ School of Biomedical Sciences Faculty of Medicine The Chinese University of Hong Kong Hong Kong SAR China; ^2^ Institute of Clinical Pharmacology Key Laboratory of Anti‐Inflammatory and Immune Medicine Ministry of Education Anhui Collaborative Innovation Center of Anti‐Inflammatory and Immune Medicine Anhui Medical University Hefei China; ^3^ CUHK‐SDU Joint Laboratory on Reproductive Genetics School of Biomedical Sciences the Chinese University of Hong Kong Hong Kong SAR China; ^4^ School of Biomedical Sciences Core Laboratory Shenzhen Research Institute The Chinese University of Hong Kong Shenzhen China; ^5^ Key Laboratory for Regenerative Medicine Ministry of Education School of Biomedical Sciences The Chinese University of Hong Kong Hong Kong SAR China; ^6^ Division of Intramural Research Eunice Kennedy Shriver National Institute of Child Health and Human Development National Institutes of Health Bethesda MD USA

**Keywords:** WRN, Werner syndrome, HGF, angiogenesis, PI3K/AKT, iPSC

## Abstract

*WRN* mutation causes a premature aging disease called Werner syndrome (WS). However, the mechanism by which WRN loss leads to progeroid features evident with impaired tissue repair and regeneration remains unclear. To determine this mechanism, we performed gene editing in reprogrammed induced pluripotent stem cells (iPSCs) derived from WS fibroblasts. Gene correction restored the expression of WRN. *WRN* ^+/+^ mesenchymal stem cells (MSCs) exhibited improved pro‐angiogenesis. An analysis of paracrine factors revealed that hepatocyte growth factor (HGF) was downregulated in *WRN* ^−/−^ MSCs. HGF insufficiency resulted in poor angiogenesis and cutaneous wound healing. Furthermore, HGF was partially regulated by PI3K/AKT signaling, which was desensitized in *WRN* ^−/−^ MSCs. Consistently, the inhibition of the PI3K/AKT pathway in *WRN* ^+/+^ MSC resulted in reduced angiogenesis and poor wound healing. Our findings indicate that the impairment in the pro‐angiogenic function of WS‐MSCs is due to HGF insufficiency and PI3K/AKT dysregulation, suggesting trophic disruption between stromal and epithelial cells as a mechanism for WS pathogenesis.

## Graphical abstract

Through correction of the Werner syndrome (WS) causative gene WRN, the paper reports a mechanism of HGF insufficiency in WS MSC and its connection with the impaired pro‐angiogenesis function. PI3K/AKT is the downregulated pathway in WS MSC.

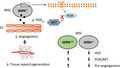

## 1 INTRODUCTION

Werner syndrome (WS) is an autosomal recessive disorder characterized by premature aging. WS is caused by genetic mutation in *WRN*, a member of the *RECQ* helicase family, and is crucial for the maintenance of genomic stability and aging (Yu et al., 1996). WS patients show early onset of many age‐associated diseases, such as premature appearance of cataracts, alopecia, osteoporosis, osteosclerosis, soft tissue calcification, hypogonadism, diabetes mellitus, and premature vascular disease (arteriosclerosis and atherosclerosis), and a high incidence of mesenchymal tumors (Oshima, Sidorova, & Monnat, 2017; Shamanna et al., 2017). The senile appearance is not apparent until the second or third decades of life. Therefore, WS is also called “adult progeria” and is distinct from classical Hutchinson–Gilford progeria syndrome (Kudlow, Kennedy, & Monnat, 2007). WS patients also display characteristic scleroderma‐like changes in the skin, such as hyperkeratosis, atrophic skin, telangiectasia, and skin ulceration on the feet and ankles. Skin ulcer is usually chronic, severe, and intractable and may lead to amputation. Clinical evidence and recent research suggest that stem cells in adults with WS can be severely affected and gradually lose their hemostasis and regenerative function over time (Muftuoglu et al., 2008; Zhang et al., 2015). However, the mechanism through which WRN loss leads to impairment of the stem cell function relevant to progeroid features and the dermatologic pathology remains unclear.

To elucidate the pathogenesis of WS stem cells during premature aging, we used reprogrammed induced pluripotent stem cells (iPSCs) from WS fibroblasts and corrected the disease‐causative gene *WRN* by using CRISPR/Cas9. Gene‐corrected *WRN* ^+/+^ WS iPSCs restored the expression of WRN. Upon differentiating into mesenchymal stem cells (MSCs), one of the most severely affected adult stem cells, *WRN* ^+/+^ MSCs, exhibited enhanced clonogenicity and improved pro‐angiogenesis. An analysis of stromal trophic factors revealed that hepatocyte growth factor (HGF) is critically downregulated in *WRN* ^−/−^ WS‐MSCs. Because HGF directly promotes mitogenesis and angiogenesis during tissue regeneration (e.g., wound healing and bone fracture repair), we hypothesize that the impaired pro‐angiogenesis of WS‐MSCs is HGF dependent. In support of this hypothesis, we performed transplantation of *WRN* ^+/+^ and *WRN* ^−/−^ MSCs in a bone defect model and a cutaneous wound model of mice and demonstrated a remarkable difference in MSC‐stimulated angiogenesis and tissue regeneration. Furthermore, the PI3K/AKT pathway was downregulated in WS‐MSCs. Our results suggest functional impairment of WS‐MSCs in angiogenesis through dysregulation of HGF expression, which may account for the poor healing of chronic ulcers and slow tissue regeneration observed in WS patients.

## 2 RESULTS

### 2.1 Genetic correction of *WRN* in WS iPSCs

**Figure 1:**
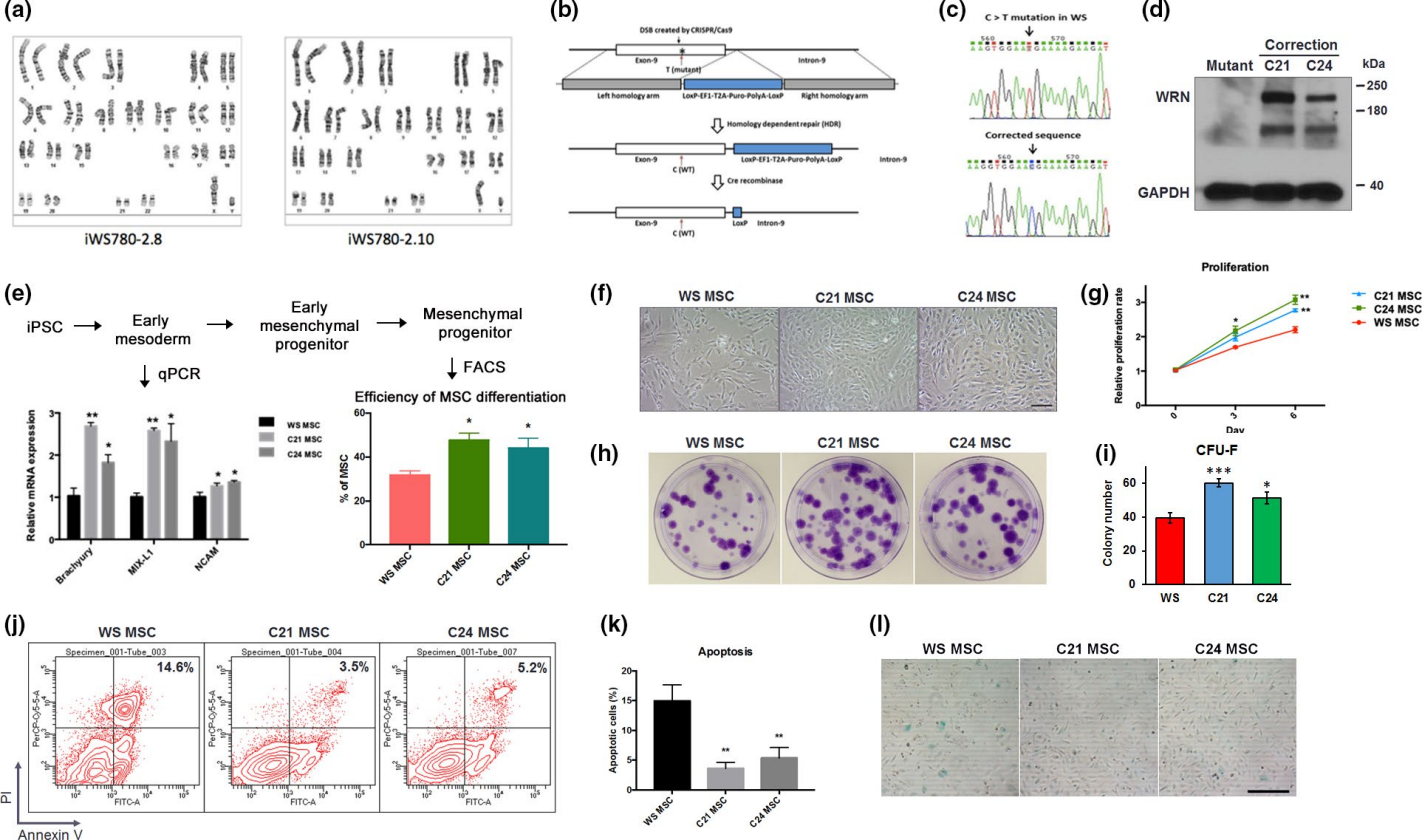
Genetic correction of *WRN* in WS iPSCs reveals impaired mesodermal differentiation of WS stem cells. (a) WS iPSCs were derived from AG00780. Two iPSC clones (iWS780‐2.8 and iWS780‐2.10) with a normal 46,XY karyotype were used for gene correction. (b) Schematic showing gene correction of *WRN* mutation in exon‐9 by homology‐dependent repair (HDR). CRISPR/Cas9 was employed to improve HDR efficiency. (c) Confirmation of gene correction through Sanger sequencing. (d) Detection of WRN in corrected iPSCs (C21 and C24) through Western blotting. (e) Differentiation of MSCs from iPSCs. Expression of early mesodermal markers (*Brachyury*, *MIX‐L1*, and *NCAM*) was measured using qPCR during mesodermal induction at day 4. Efficiency was determined by the expression of CD90^+^/CD105^+^/CD73^+^ and CD45^−^/CD34^−^/CD11b^−^/CD19^−^/HLA‐DR^−^ immunophenotype through FACS after 21 days of differentiation. (f, g) Phase‐contrast images of WS‐, C21‐, and C24‐derived MSCs and their proliferation rates in culture. (h, i) CFU‐F assay showed increased clonogenicity in C21 and C24 MSCs. (j, k) Cell apoptosis of WS, C21, and C24 MSCs under normal culture conditions. (l) Senescence‐associated β‐galactosidase (SA‐β‐gal) staining showed reduced senescence in C21 and C24 MSCs. Scale bar: 100 μm in (f) and (l); *p < .05; **p < .01; ***p < .001

We previously reprogrammed a number of WS fibroblasts carrying different mutations in *WRN* (Cheung et al., 2014). Loss of WRN does not prevent epigenetic reprogramming or the maintenance of telomeres and pluripotency in iPSCs (Cheung et al., 2014; Shimamoto et al., 2015). However, upon differentiation into multipotent MSCs, premature senescence and reduced replicative potential were observed, similar to the properties of WS fibroblasts. These intrinsic properties make WS‐MSCs unsuitable for cell therapy. To rescue the genetic defect, we screened nine iPSC clones from a WS patient (AG00780, acquired from Coriell Institute) carrying a typical nonsense C > T mutation in exon‐9 of *WRN* for genomic integrity (Table S1). The point mutation causes premature translational termination (p.R369X) at the region between the exonuclease and helicase of WRN, resulting in the expression of a truncated protein lacking helicase activity and the nuclear location signal. Only two iPSC clones (iWS780‐2.8 and iWS780‐2.10) carried a normal 46 XY karyotype (Figure 1a). Through homology‐dependent repair (HDR), the point mutation was corrected using a targeting construct and a CRISPR/Cas9 expression vector (Figure 1b). We randomly picked two clones, designated as C21 and C24, with both alleles targeted by Cas9 and corrected by HDR, for subsequent studies. We confirmed the gene correction through genomic DNA and mRNA sequencing (Figure 1c). Western blot analysis indicated the expression of WRN with the expected molecular size in C21 and C24 (Figure 1d).

### 2.2 Impaired mesodermal differentiation in WS stem cells

Because studies have reported the recurrence of premature senescence in mesoderm‐derived cells but not in pluripotent iPSC or ESC or neural stem cells, we differentiated C21 and C24 (*WRN* ^+/+^) and the isogenic mutant WS iPSCs (*WRN* ^−/−^) to MSCs (Figure S1). MSCs differentiated from iPSCs were sorted by a set of MSC markers (positive for CD90, CD105, and CD73 and negative for CD34, CD11b, CD19, CD45, and HLA‐DR). As revealed by marker expression, WS iPSCs differentiated into MSCs at a relatively lower efficiency than C21 and C24 iPSCs (Figure 1e). Examination of early mesodermal markers at day 4 in the differentiation protocol revealed lower levels of *Brachyury*, *MIX‐L1*, and *NCAM* transcripts (Figure 1e), suggesting that mesodermal induction is already impaired at the early stages of differentiation. Gene correction of *WRN* may not only enhance the differentiation potential but also the self‐renewal of stem cells. Gene‐corrected MSCs exhibited a higher proliferation rate and enhanced clonogenicity **(**Figure 1f‐i) and no premature senescence or increased apoptosis rate that is seen in uncorrected (WS) MSCs (Figure 1j‐l).

**Figure 2:**
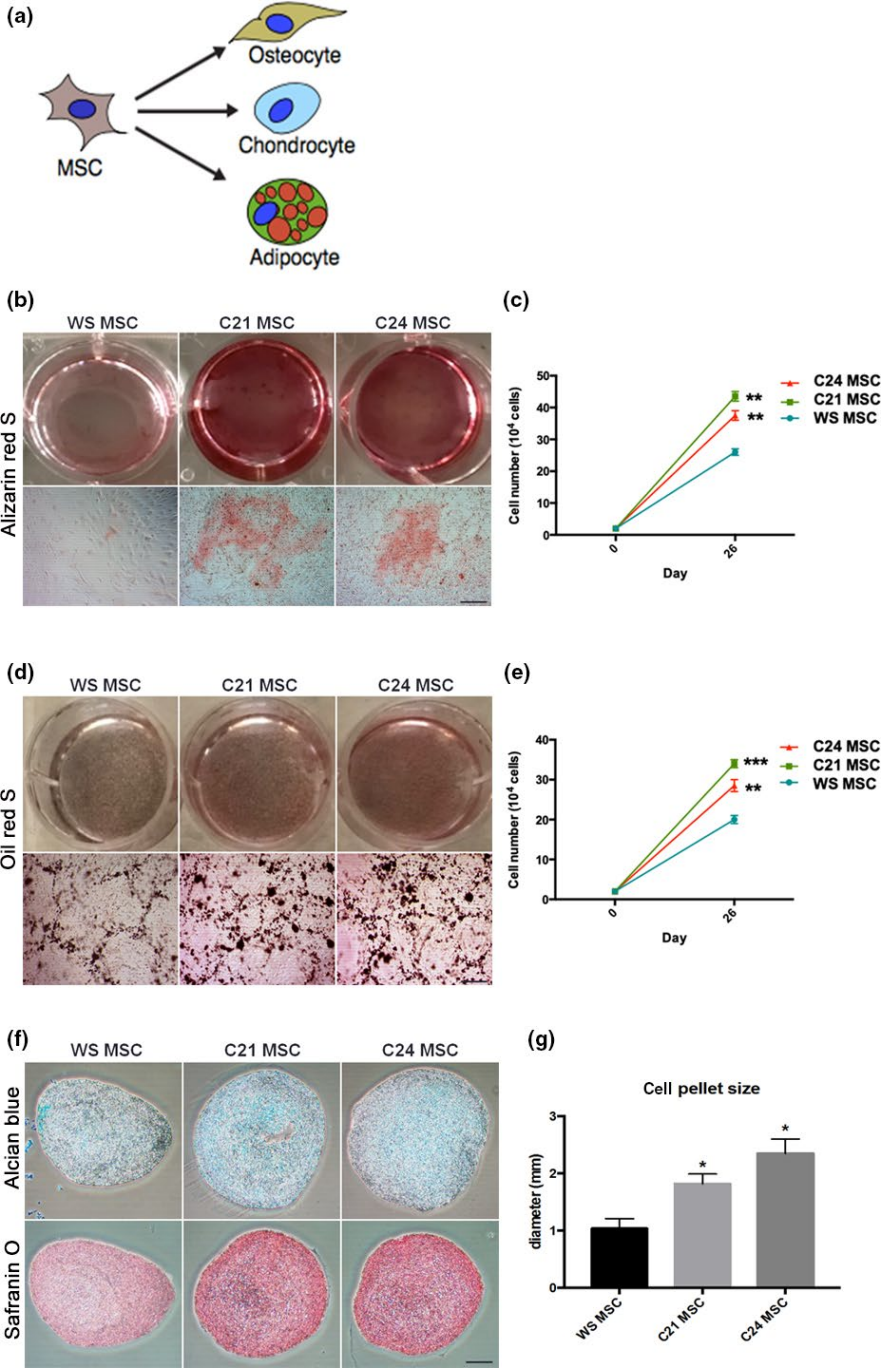
Impaired trilineage differentiation in WS‐MSCs. (a) Trilineage differentiation was performed to compare the MSC differentiation potential before and after gene correction. (b, c) Alizarin red S staining was performed for the differentiation of osteocytes. Total cell number at the end of the differentiation was counted. (d, e) Oil red S staining was performed for the differentiation of adipocytes. Total cell number at the end of the differentiation was counted. (f, g) Alcian blue and Safranin‐O staining were performed for the differentiation of chondrocytes. Cell pellet size at the end of chondrocyte differentiation was shown. Scale bar: 100 μm in (b) and (d), 300 μm in (f). *p < .05; **p < .01; ***p < .001

We next tested whether C21/C24 and WS‐MSCs could be differentiated into adipocytes, osteocytes, and chondrocytes (Figure 2a). For osteogenic differentiation, WS‐MSCs demonstrated impaired differentiation potential with less osteocytes formed by the end of differentiation (Figure 2b‐c). For adipogenic differentiation, WS‐MSCs showed a slight difference in oil droplet formation by the end of adipocyte formation (Figure 2d,e). For chondrogenic differentiation, Safranin‐O staining indicated impaired cartilage formation in WS‐MSCs (Figure 2f). The cell mass of WS chondrocytes was also lower (Figure 2g). These results demonstrated that WS stem cells exhibit reduced differentiation potential when they are induced into specific mesodermal lineages.

### 2.3 Gene‐corrected WS‐MSCs exhibit enhanced pro‐angiogenic function

**Figure 3:**
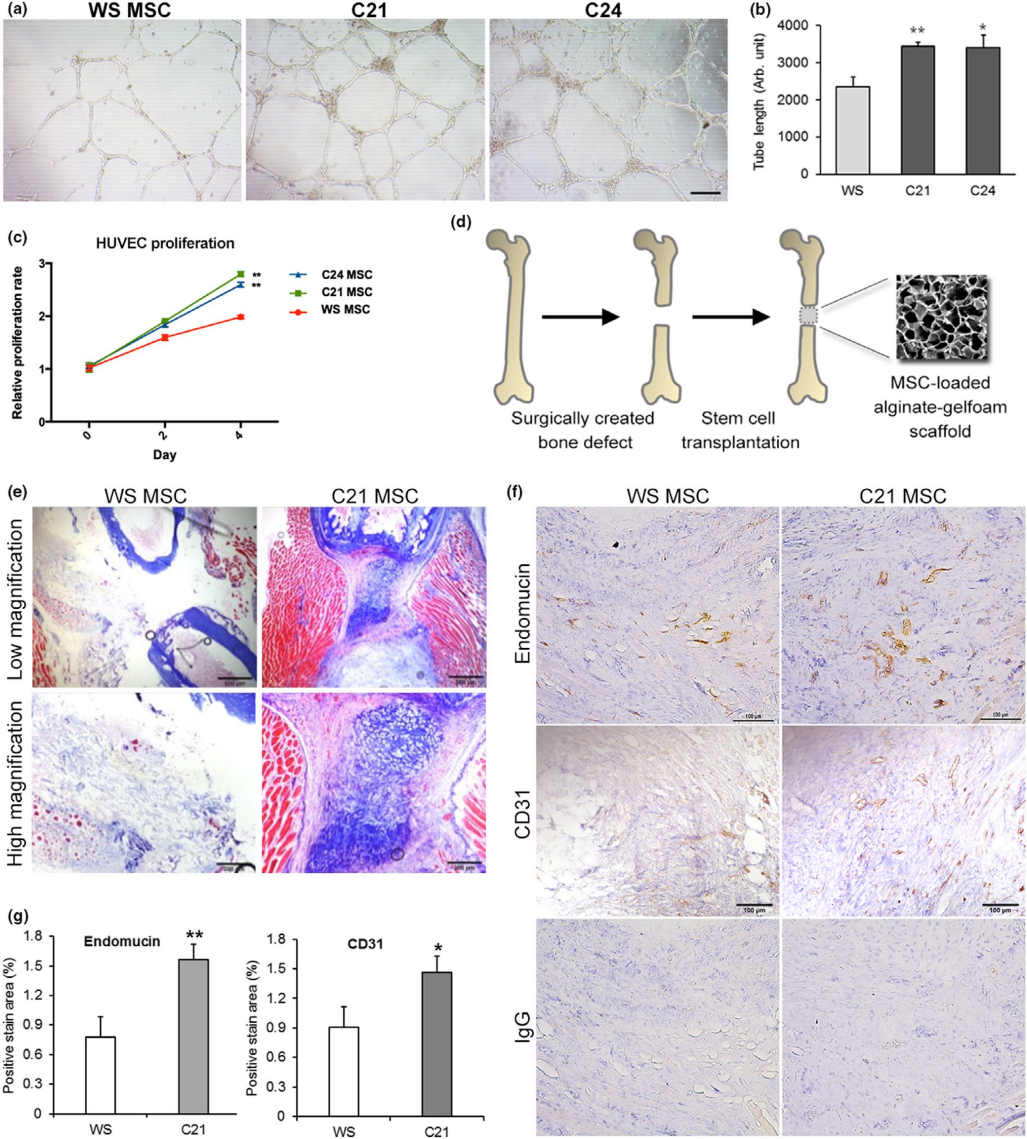
Gene‐corrected WS‐MSCs demonstrate enhanced pro‐angiogenic function. (a, b) Tube formation assay showed differential pro‐angiogenic activity by using HUVEC exposed to conditioned medium (CM) collected from WS and C21/C24 MSCs. (c) Mitogenesis of HUVEC when cells were stimulated with WS or C21/C24 MSC CM. (d) Schematic of the bone defect model for studying angiogenesis in vivo. A femur segmental bone defect was created through surgery in immunodeficient mice followed by immediate transplantation of human MSCs incorporated in 3D alginate‐gelfoam. Bone tissues were harvested 2 weeks after transplantation. (e) Masson's trichrome staining revealed regeneration of soft tissue (mineralized collagen was stained as blue, whereas muscle tissue was stained as red) in the bone defect area. (f, g) Increased expression of angiogenesis markers endomucin and CD31 indicated through immunohistochemistry. Scale bar: 100 μm in (a) and (f); 500 μm or 200 μm in (e). *p < .05, **p < .01

Adult MSCs promote tissue regeneration and repair by promoting angiogenesis. We thus investigated whether gene‐corrected MSCs exhibited improved pro‐angiogenic function. Using the HUVEC tube formation assay, we found that conditioned medium (CM) collected from C21 and C24 MSCs exhibited enhanced pro‐angiogenic activity compared with CM from WS‐MSCs (Figure 3a). Moreover, the CM of C21 and C24 MSCs exerted enhanced mitogenic and motogenic effects on HUVEC (Figure 3c, Figure S2). These data suggest that gene correction increases the paracrine effect on stimulating HUVEC tubulogenesis, mitogenesis, and motogenesis, which is indicative of an improved pro‐angiogenic function of MSCs.

### 2.4 Gene correction improves angiogenesis in vivo

Physiological angiogenesis was simulated using a femur segmental bone defect model (Wan et al., 2008). In this model, equal numbers of WS or C21 MSCs preloaded in a 3D alginate‐gelfoam scaffold were transplanted into the surgically created bone defect in adult mice (Figure 3d). Collagen formation and bone regeneration were clearly indicated through Masson's trichrome staining. In the WS‐MSC transplant group, less collagen deposition (stained as blue) was found, and bone regeneration was poor (Figure 3e). Two weeks after transplantation, angiogenesis at the bone defect area was detected through immunohistochemistry. Staining of endomucin and CD31, two markers of vessels, indicated neovascularization at the bone defect area (Figure 3f,g). These data support that gene correction of *WRN* improves the pro‐angiogenic function of MSCs and enhances tissue regeneration in vivo.

### 2.5 Downregulation of HGF in WS‐MSC

**Figure 4:**
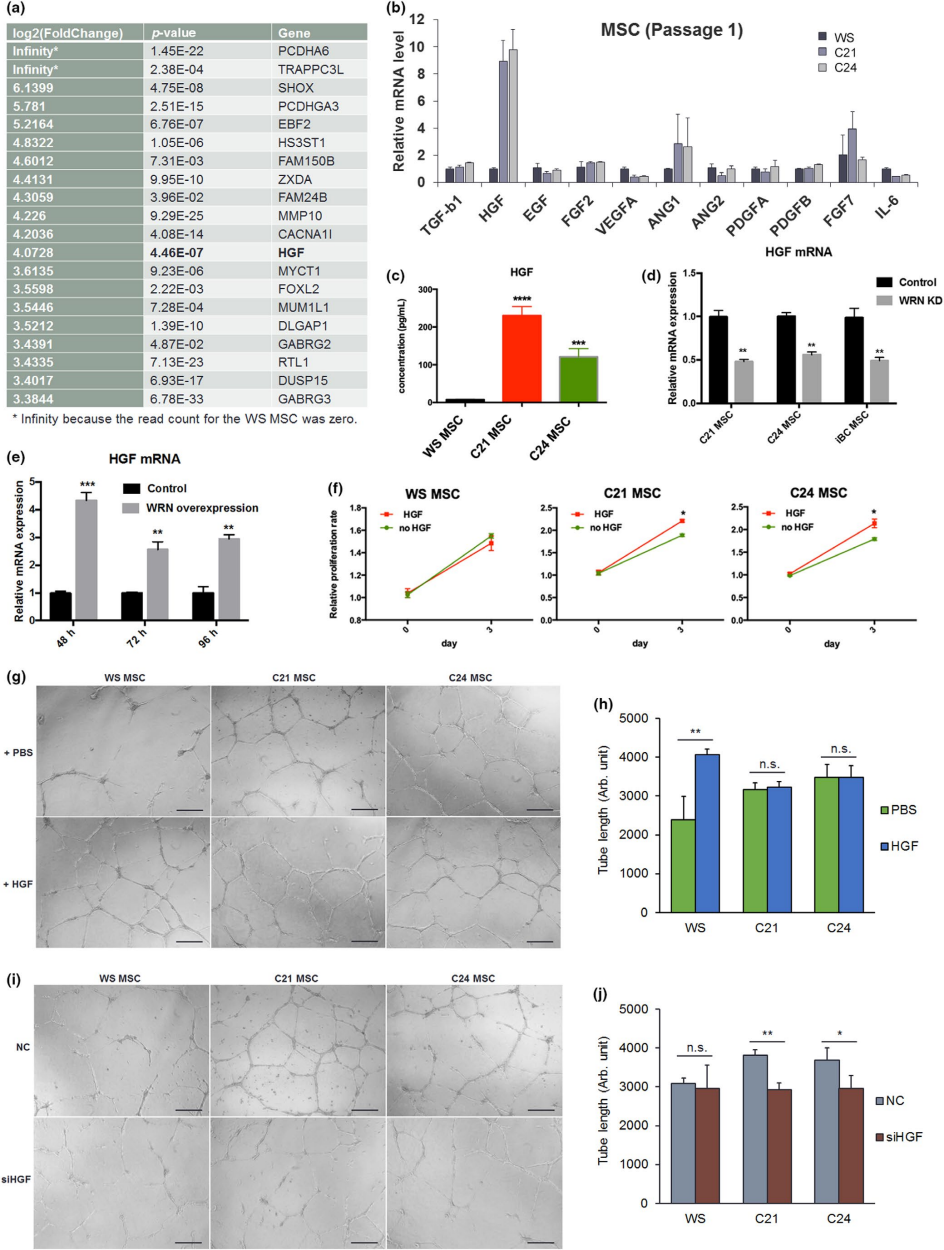
Downregulation of HGF in WS‐MSCs results in impaired pro‐angiogenic activity. (a) The top 20 upregulated genes in C21/C24 MSCs. (b) Analysis of MSC‐secreted paracrine factors in WS and C21/C24 MSCs through RT‐qPCR. (c) Secreted HGF in conditioned medium (CM) was examined through ELISA. (d) Knockdown of WRN in normal (iBC) or gene‐corrected (C21/C24) MSCs resulted in downregulation of HGF expression. (e) Overexpression of wild‐type WRN in WRN‐null WS‐MSCs resulted in upregulation of HGF expression. (f) Addition of recombinant HGF protein to medium promoted proliferation of C21/C24 but not WS‐MSCs. (g, h) Addition of recombinant HGF protein to WS‐MSC CM promoted tube formation of HUVEC. (i, j) Knockdown of HGF in C21/C24 MSCs resulted in reduced angiogenesis of HUVEC. Scale bar: 100 μm in (g) and (i). *p < .05; **p < .01; ***p < .001; n.s. not significant

Mesenchymal stem cells promote angiogenesis by secreting a number of trophic factors and cytokines through the paracrine effect (Ma et al., 2014). To determine whether the poor pro‐angiogenic function of WS‐MSCs is because of changes in paracrine factors, we compared the transcriptional profiles of WS and gene‐corrected MSCs by using RNA‐seq. The most significantly and highly upregulated genes in C21/C24 are listed in Figure 4a. Among these, HGF is the only MSC‐secreted paracrine trophic factor related to angiogenesis and tissue regeneration. Therefore, we validated other MSC‐secreted paracrine factors through RT‐qPCR and ascertained that HGF was significantly downregulated in WS‐MSCs (Figure 4b). HGF is a stromal cell‐secreted growth factor known to stimulate angiogenesis, mitogenesis, motogenesis, and morphogenesis (Trusolino et al., 2010). We confirmed the differential expression of HGF by measuring secreted HGF protein in CM by using ELISA (Figure 4c). To clarify the role of WRN in HGF expression, we depleted WRN in WRN^+/+^ MSCs by siRNA. HGF mRNA was decreased accordingly (Figure 4d). In primary human MSCs (umbilical cord‐derived), siRNA‐mediated knockdown of WRN or pharmacological inhibition of WRN activity also resulted in HGF reduction (Figure S3). Furthermore, transfection of wild‐type WRN expression plasmids in WS‐MSCs increased HGF expression (Figure 4e). Thus, downregulation of HGF in WS‐MSCs appears to be WRN‐dependent and not cell line‐specific. We examined the levels of WRN and HGF transcripts in different passages of primary MSCs and found a high positive correlation (*R* = .872) between WRN and HGF levels (Figure S3e). Moreover, the addition of HGF recombinant protein to culture medium promoted the proliferation of C21 and C24 MSCs but not that of WS‐MSCs (Figure 4f). Collectively, our data suggest that downregulation of HGF can contribute to the poor pro‐angiogenic function in WS‐MSCs. Next, we tested the effects of HGF on HUVEC‐mediated angiogenesis in vitro. Recombinant HGF protein added to the CM of WS‐MSCs increased HUVEC angiogenesis (Figure 4g,h) and migration/invasion (Figure S4), whereas knockdown of HGF in C21/C24 MSCs reversed these effects (Figure 4i,j). In addition to the WS iPSC model, we generated a WS ESC model by knocking out WRN in H1 ESCs. Similarly, ESC‐derived MSCs also demonstrated the downregulation of HGF and impaired pro‐angiogenesis in WRN‐deficient cells (Figure S7). Taken together, these results indicate that the downregulation of HGF in WS impairs the pro‐angiogenic function of MSCs.

### 2.6 Gene‐corrected WS‐MSCs promote cutaneous wound healing in diabetic physiology

**Figure 5:**
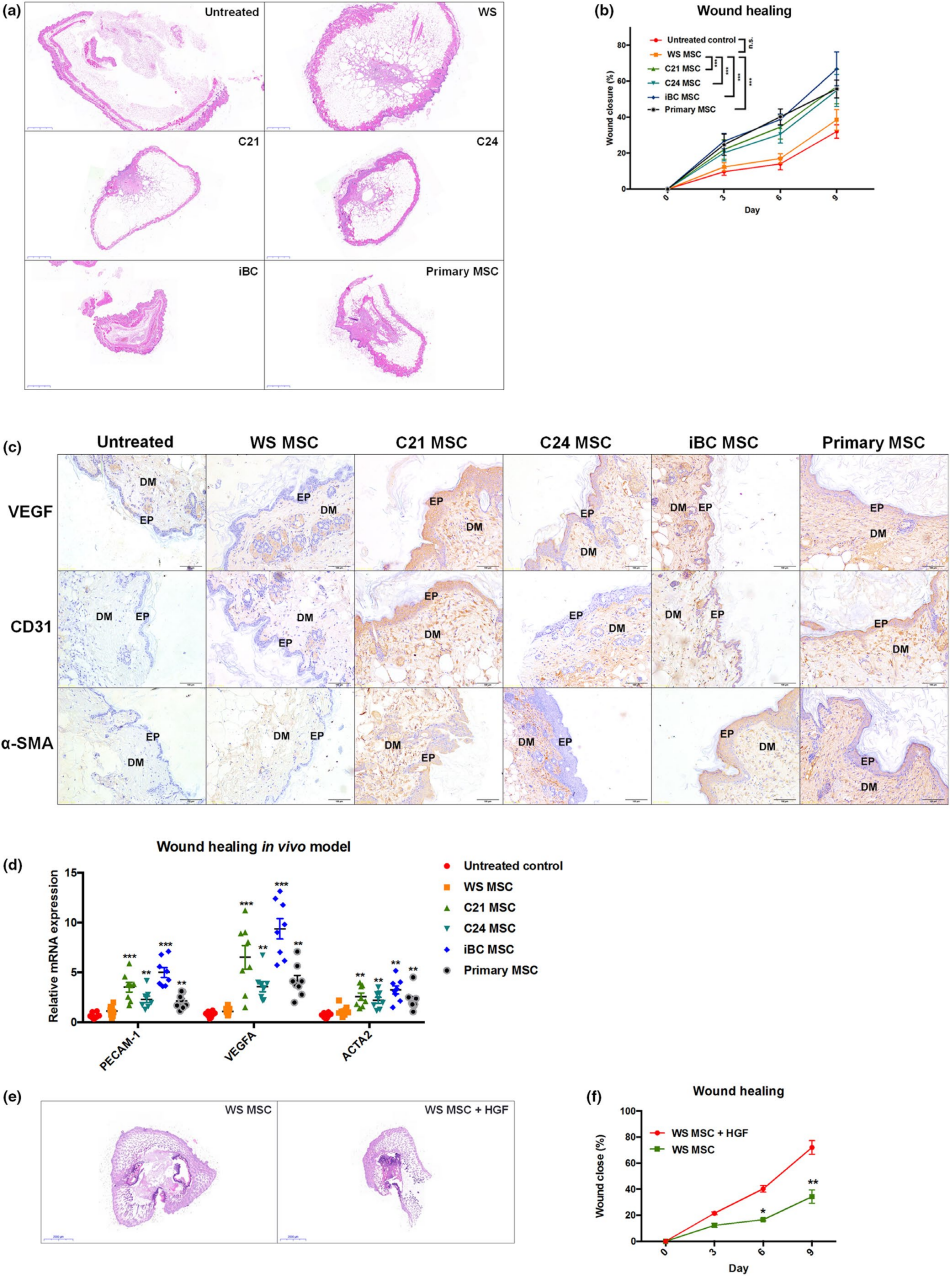
Gene‐corrected WS‐MSCs promote cutaneous wound healing in diabetic condition. (a) An incisional cutaneous wound with a diameter of 4.5 mm was created in diabetic mice (*Lepr^db^*). MSCs were transplanted onto the wound bed immediately. Skin tissues were harvested and analyzed using H&E staining. (b) The wound healing process was monitored during the first 9 days after MSC transplantation. (c) Immunohistochemical analysis of the markers for angiogenesis (VEGF and CD31) and myofibroblasts (α‐SMA) in WS and C21/C24‐treated wounds. Normal iPSC‐derived (iBC) MSCs and primary MSCs were included as additional controls. DM: dermis; EP: epidermis. (d) Quantification of markers in (c) through qPCR. (e, f) Addition of recombinant HGF to WS‐MSCs resulted in improved and accelerated wound healing. Scale bar: 2,000 μm in (a) and (e); 100 μm in (c). *p < .05; **p < .01; ***p < .001; n.s. not significant

WS patients frequently experience chronic skin ulcers, which are difficult to heal. In addition, the recurrence rate is considerably higher in WS patients than in nonsyndromic patients (Nakagami et al., 2017). Skin atrophy and type‐2 diabetes are the common phenotypes of WS, further challenging wound healing and tissue regeneration. MSCs have been demonstrated to enhance wound healing through differentiation and angiogenesis (Wu, Chen, Scott, & Tredget, 2007). To demonstrate whether a deficiency of HGF expression in WS‐MSCs results in impaired therapeutic potential of MSCs, we transplanted the same numbers of WS, C21, and C24 MSCs onto surgically created cutaneous wounds in diabetic mice (7–8‐week old, female *Lepr* ^db^ mice; Figure S5a‐c). Normal iPSC‐derived MSCs (iBC) and primary human MSCs were included as additional controls. On stem cell treatment, C21/C24 or iBC/primary MSCs promoted wound healing at a faster rate than WS‐MSCs or untreated controls (Figure 5a,b). The difference between untreated and vehicle controls was not significant (Figure S5d,e). Notably, the addition of HGF recombinant protein significantly enhanced the wound healing rate more than WS‐MSCs alone did (Figure 5e,f). MSCs can improve wound healing outcomes by promoting angiogenesis and stimulating tissue repair as well as other modulatory effects (e.g., immunomodulation; Ma et al., 2014). Histological examination of the skin biopsy specimens after 9 days of MSC transplantation revealed abundant CD31‐positive staining and thicker epidermal/dermal layers in the C21/C24 MSC‐treated skin than in the untreated or WS‐MSC‐treated skin (Figure 5c,d). High levels of VEGF, another marker for angiogenesis, were also detected in the C21/C24 MSC‐treated wound than in the WS‐MSC‐treated wound. In the granulation tissue, fibroblasts are differentiated into myofibroblasts, which stimulate the expression of α‐SMA (also known as ACTA2) and help secrete ECM proteins. α‐SMA staining demonstrated increased myofibroblast activation in C21/C24‐treated wounds (Figure 5c). Remarkably, the addition of HGF to WS‐MSCs enhanced the expression of all these markers in healing wounds (Figure S5f).

### 2.7 Role of PI3K/AKT signaling in regulating HGF

**Figure 6:**
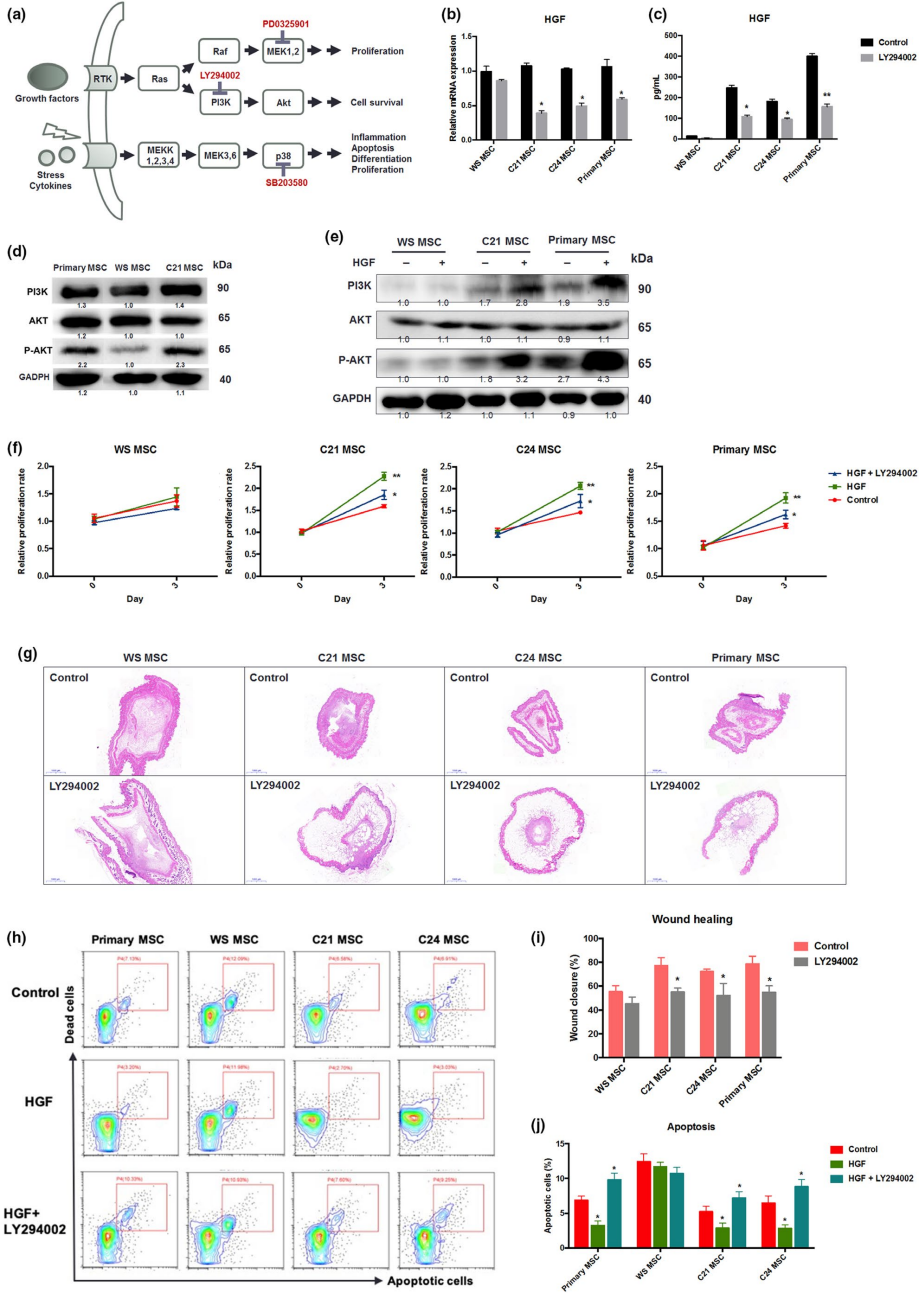
Role of PI3K/AKT signaling pathway in regulating HGF and wound healing. (a) Schematic showing the major signaling pathways (Ras/Raf, PI3K/AKT, and MAPK/p38) altered in WS‐MSCs. PD0325901, LY294002, and SB203580 were used to inhibit these pathways, respectively. (b, c) Expression of HGF in response to LY294002 treatment. HGF was measured using RT‐qPCR and ELISA. (d) Western blot analysis of the PI3K/AKT pathway in WS, C21, and primary MSCs. P‐AKT is the phosphorylated form of AKT due to PI3K activation. (e) Western blot analysis of the PI3K/AKT pathway when MSCs were treated with HGF recombinant protein. (f) Effect of HGF or HGF plus LY294002 treatment on cell growth of MSCs. (g, i) Targeting the PI3K/AKT pathway compromises MSC‐mediated wound healing. Cutaneous wound was treated with MSCs with or without LY294002. The wound area was analyzed after 7 days. (h, j) Effect of HGF or HGF plus LY294002 treatment on MSC apoptosis. Dead cells were stained using PI, whereas apoptotic cells were stained using Annexin V. Scale bar: 1,000 μm. *p < .05; **p < .01

How the loss of WRN results in HGF deficiency is elusive. We analyzed signaling pathways that altered between WS and C21/C24 MSCs by using RNA‐seq data. On the top of pathways, the Ras/Raf, PI3K/AKT, and MAPK/p38 signaling pathways were enriched and relevant to MSC functionality (Figure S6a). To examine which pathway is critical for HGF expression, we used small molecules to block each pathway (PD0325901 for Ras/Raf/MEK, SB203580 for MAPK/p38, and LY294002 for PI3K/AKT; Figure 6a). Although all tested inhibitors could suppress HGF expression in *WRN* ^+/+^ cells, only LY294002 (a PI3K inhibitor) showed a differential effect (Figure 6b,c). WS‐MSCs appeared less sensitive to LY294002 than to C21/C24. Western blot analysis revealed a reduction in the levels of phosphorylated AKT (p‐AKT) protein in WS‐MSCs (Figure 6d), suggesting that the PI3K/AKT signaling pathway is impaired. In MSCs, PI3K/AKT signaling can be activated by different growth factors including TGF‐α and HGF itself, leading to increased cell survival, proliferation, migration, and pro‐angiogenic activity (Forte et al., 2006; Wang et al., 2009). We found that PI3K/AKT signaling could be activated by treating *WRN* ^+/+^ MSCs with HGF (Figure 6e) and lead to increased proliferation (Figure 6g,h). The growth factor‐induced mitogenic effect was counteracted by LY294002 treatment. However, PI3K/AKT signaling in WS‐MSCs appeared inert, and HGF could not activate AKT and stimulate cell proliferation (Figure 6e,f). We further investigated whether PI3K/AKT is critical for WS‐MSC survival. Under normal culture conditions (20% oxygen, 10% serum with low glucose medium), WS‐MSCs exhibited a higher rate of apoptosis. The addition of HGF promoted MSC survival, whereas the addition of LY294002 reduced cell survival in *WRN* ^+/+^ but not in *WRN* ^−/−^ cells (Figure 6j,l). In line with a previous report (Wang et al., 2009), TGF‐α was also able to stimulate HGF expression, which was suppressed by LY294002 in *WRN* ^+/+^ but not in *WRN* ^−/−^ cells (Figure S6b,c). Taken together, these results suggest that inactive PI3K/AKT signaling in WS‐MSCs compromises cell proliferation and survival.

Last, we evaluated the role of the PI3K/AKT pathway in the therapeutic potential of stem cells. The inhibition of PI3K/AKT signaling by LY294002 in *WRN* ^+/+^ MSCs resulted in a poor therapeutic effect, as indicated by retarded wound healing (Figure 6i,k). WS‐MSCs, regardless of the presence of an inhibitor, showed poor wound healing‐promoting effect. All these results suggest that the PI3K/AKT pathway is critical for mediating the (trophic) function of MSCs, whereas in WS stem cells, this pathway is downregulated or desensitized, which accounts for the poor therapeutic outcome of WS stem cells.

## 3 DISCUSSION

The pathogenesis of premature aging in WS has been previously investigated largely using nonhuman model organisms (e.g., yeast, fly, worm, and mouse; Lautrup et al., 2019) or human tissue biopsies (e.g., skin fibroblasts and lymphoblasts; Mazzarello, Ferrari, & Ena, 2018). Clinical reports on WS provide additional pathological insights into the mechanistic origin of the disease (Goto, Ishikawa, Sugimoto, & Furuichi, 2013). However, why the defect of the causative gene *WRN* causes impaired regenerative capacity and overall senescence is intriguing. One explanation for this phenomenon is the accelerated depletion of the adult stem cell pool (Cheung, Pei, & Chan, 2015; Zhang et al., 2015). The availability of a human iPSC/ESC model provides a new approach to test such hypotheses. There is, however, a lack of molecular association between WRN loss and the impaired tissue repair and regeneration, which involve adult stem cells. Here, we described that genetic correction of *WRN* in WS iPSCs conferred the stem cells with enhanced differentiation potential, clonogenicity, survival, and pro‐angiogenic function upon differentiation into MSCs. Gene‐corrected MSCs also displayed enhanced therapeutic potential in promoting cutaneous wound healing in diabetic mice. Using this approach, HGF was identified to be a key paracrine factor responsible for MSC‐mediated angiogenesis and regeneration. The significance of HGF insufficiency in association with WS pathophysiology has not been revealed before. HGF is indispensable for embryonic liver development, whereas in adults, it is important for tissue repair and regeneration by promoting cell proliferation, survival, and angiogenesis. Conditional knockout of HGF/Met in different organs results in developmental or regenerative defects of the epithelium (Kato, 2017; Nejak‐Bowen, Orr, Bowen, & Michalopoulos, 2013). In our study, WS‐MSCs poorly promoted cutaneous wound healing in diabetic mice, whereas the addition of HGF reinforced this effect. Our finding is consistent with a previous finding that the ablation of Met in keratinocytes results in impaired re‐epithelialization during wound repair (Chmielowiec et al., 2007).

The paracrine action of MSCs is essential for cell therapy and tissue engineering. MSCs secrete a number of cytokines (e.g., VEGF, FGF, HGF, and PDGFA/B) to recruit and activate/stabilize other cells (e.g., fibroblasts, keratinocytes, and endothelial cells) and promote cell migration, proliferation, survival, and morphogenesis (Madrigal, Rao, & Riordan, 2014; Spees, Lee, & Gregory, 2016). HGF‐null MSCs are unable to repair ischemic limbs (Nakagami et al., 2005). The PI3K/AKT signaling pathway has been demonstrated to promote the secretion of trophic factors including HGF (Forte et al., 2006). Genetically modified MSCs with enhanced Akt signaling improves infarcted myocardium repair (Mangi et al., 2003). We incidentally revealed the relevance of the PI3K/AKT pathway in WS. The PI3K/AKT signaling was exceptionally inactive in WS‐MSCs, and thus, the PI3K inhibitor LY294002 had no significant inhibitory effect. However, the inhibition of PI3K/AKT in *WRN* ^+/+^ MSCs resulted in the downregulation of HGF and reduced wound closure in *WRN* ^+/+^ MSCs. Consistent with other findings, stimulators of PI3K/AKT signaling, such as SDF‐1, miR‐126, and angiopoietin‐1, are pro‐angiogenic in nature (Chen & Zhou, 2011; Piao et al., 2010; Tang et al., 2009). The mechanism by which WRN loss results in desensitized PI3K/AKT signaling and reduced HGF expression remains elusive, although we have confirmed the relationship using a gene rescue experiment in which the expression of wild‐type WRN in WS cells increased HGF levels. Dissecting the global DNA occupancy by WRN using WRN‐ChIP might be noteworthy. Another possible explanation for this phenomenon is the increased cellular stress as a result of WRN loss (Szekely et al., 2005). Although both in vitro and in vivo angiogenesis assays suggest reduced angiogenesis outcomes in WS‐MSCs, the paracrine effect would not be the only factor. WS cells are less proliferative, which may result in reduced cell numbers after transplantation. Moreover, WS‐MSCs are known to survive poorly in vivo (Zhang et al., 2015), which may account for the poor therapeutic effect in promoting skin and bone repairs. Apart from HGF, we found other downregulated factors related to angiogenesis in WS (Figure 4a). For instance, MMP10 is a matrix metalloproteinase that digests extracellular matrix proteins to facilitate angiogenesis and tissue remodeling. The mechanism through which WRN loss causes the downregulation of other factors must be further elucidated. Nevertheless, the current study suggests that WS‐MSCs cannot promote angiogenesis as well as normal MSCs and that reduced HGF production is independent of cell proliferation or survival.

A question that remains to be elucidated regarding the pathogenesis of WS is the cell lineage selectivity of the disease. The mesenchymal lineage and connective tissue (e.g., fibroblasts) are overall more severely affected, which might be attributed to the premature senescence phenotype. However, premature senescence in vivo has been challenged by recent findings using dermal fibroblasts from autopsied WS patients (Ibrahim et al., 2016; Tokita et al., 2016). From our study, although premature senescence of WS‐MSCs (compared with gene‐corrected MSCs) could be observed in late passages, HGF deficiency was observed when MSCs were immediately differentiated from mesoderm precursors. Therefore, HGF deficiency appears to be WRN‐dependent but not senescence‐ or critically short telomere‐driven. In line with the notion proposed in a recent book chapter reviewed by Oshima, Hisama, and Monnat Jr. suggesting that the WS pathogenesis can be a result of “disruption of trophic or regulatory interactions required to maintain adjacent epithelial or stromal cells and tissue architecture,” our work provided the first experimental evidence to support this hypothesis.

In summary, our results explain some of the clinical phenotypes of WS‐poor angiogenesis in chronic wound outcomes in refractory ulcers and poor tissue repair and regeneration. It is also a critical factor for ischemia due to impaired vessel formation or premature vascular aging, which is associated with atherosclerosis and arteriosclerosis. Our findings suggest a therapeutic intervention that involves the activation of PI3K/AKT signaling or expression of pro‐angiogenic factors, such as HGF, to ameliorate the symptoms by enhancing the therapeutic outcomes of stem cell‐based therapy.

## CONFLICT OF INTEREST

The authors declare that the research was conducted in the absence of any commercial or financial relationships that could be construed as a potential conflict of interest.

## AUTHOR CONTRIBUTIONS

HC conceived of the study, participated in its design and coordination, and drafted the manuscript. JT carried out most of the experiments. CW and FZ carried out the bone defect transplantation and data analysis. LC, PL, YT, and GL participated in experimental work. OR and WC participated in the design and coordination of the study.
