# Corrigendum: Genetic correction of Werner syndrome gene reveals impaired pro‐angiogenic function and HGF insufficiency in mesenchymal stem cells

**DOI:** 10.1111/acel.13227

**Published:** 2020-09-30

**Authors:** Jiajie Tu, Chao Wan, Fengjie Zhang, Lianbao Cao, Patrick Wai Nok Law, Yuyao Tian, Gang Lu, Owen M. Rennert, Wai‐Yee Chan, Hoi‐Hung Cheung

In the original publication of J. Tu et al., (2020), the Materials and Methods section was not included. The missing section is shown below.

## 4 MATERIALS AND METHODS

### 4.1 Gene correction of WS iPSC

We acquired the WS fibroblasts from Coriell Institute. We derived WS iPSC (iWS780 clone 2.10) from WS fibroblast AG00780. This patient carries a 1336C>T mutation in exon‐9 of *WRN*, causing premature translational termination (p.R369*) of WRN protein. We designed a guide RNA sequence (gatgtacttggaaataaaag) near the mutation and cloned into the CRISPR/Cas9 plasmid PX458 (Addgene#48138). We constructed the targeting vector by cloning the left homology arm (including the entire exon‐9) and right homology arm (intron‐9) into an HR vector HR100PA (SBI). We changed the targeting sequence on the left homology arm by synonymous substitution to avoid secondary gene editing by the gRNA. We transfected the CRISPR/Cas9 plasmid and the linearized targeting vector into WS iPSC by nucleofection. Puromycin‐resistant colonies were expanded on MEF. We examined successfully corrected clones by detecting expression of WRN protein by Western blot. We confirmed correct homologous recombination by sequencing the genomic DNA region covering the entire exon‐9 and part of intron‐9. We confirmed correct mRNA splicing by sequencing the cDNA of the entire *WRN* open reading frame. To generate an ESC model of WS, we transfected human H1 ESC line with the same gRNA shown above by nucleofection. A non‐specific control gRNA was used to derive the control line. After colony formation from single cells (GFP‐sorted), we screened individual clones by Western blot using anti‐WRN antibody. WRN‐null clones were validated by genomic sequencing across exon‐9.

### 4.2 Cell culture and differentiation

We cultured iPSC on irradiated MEF using iPSC medium (KO‐DMEM containing 20% KSR, 1% NEAA, 1% GlutaMAX, 0.1 mM 2‐Mercaptoethanol, 1% P/S, 10 ng/mL bFGF). Medium was changed every day until being passed with Collagenase IV. We cultured iPSC on matrigel (Corning) using mTeSR1 medium (Stemcell Technologies) before differentiation. Medium was changed every day until being passed with Dispase. To differentiate iPSC into MSC, iPSC cultured in feeder‐free condition was dissociated into single cells and plated on matrigel with mTeSR1 medium containing 10 μM Y‐27632 (Selleckchem). We induced early mesodermal progenitor cells using STEMdiff‐ACF Mesenchymal Induction Medium (Stemcell Technologies), according to the manufacturer's instruction. On day 4, medium was switched to MesenCult‐ACF medium (Stemcell Technologies) to promote differentiation into MSC. Cells were passed when becoming confluent and plated on MesenCult‐ACF Attachment Substrate. By day 21, cells formed typical MSC morphology. We used the BD Stemflow hMSC Analysis Kit (BD Biosciences) to sort cells with CD105^+^/CD73^+^/CD90^+^ and CD45^−^/CD34^−^/CD11b^−^/CD19^−^/HLA‐DR^−^ immunophenotype by FACS. The phenotypic signature is described by The Mesenchymal and Tissue Stem Cell Committee of the International Society for Cellular Therapy (ISCT). We gated CD45/CD34/CD11b/CD19/HLA‐DR negative cells by PE, followed by gating CD90, CD105, CD73 positive cells by FITC, PerCP‐Cy5 and APC respectively. The resulting gated cells were divided by the total cell number before gating and represented as “efficiency of MSC differentiation”. We tested the multipotency of MSC by trilineage differentiation using induction media purchased from Stemcell Technologies and Gibco. At the end of the trilineage differentiation, total cell number was counted (osteogenesis and adipogenesis) or the cell pellet size (chondrogenesis) was measured. Primary MSC was purchased from ScienCell.

### 4.3 Colony‐forming unit‐fibroblast (CFU‐F) assay

For CFU‐F assay, we seeded 150 single cells of first‐passage MSC on 10‐cm dish. Cells are allowed to form colonies in MSC medium (Low‐glucose DMEM containing 10% FBS) for 3 weeks. At the end of the experiment, colonies were stained with 0.5% Crystal Violet and photographed.

### 4.4 Cell proliferation assay

5 × 10^3^ cells (MSC or HUVEC) were seeded in 96‐well plate for 2–6 days. Cell proliferation was measured using Cell Counting Kit‐8 (DOJINDO) according to the manufacturer's protocol. A growth curve was plotted by showing the relative cell number comparing to day 0.

### 4.5 In vitro angiogenesis assays

We coated matrigel (BD) in cold 24‐well plate (240 μl for each well) and congealed the matrigel in 37°C tissue culture incubator for 20 min. 0.5 × 10^5^ HUVEC cells suspended in MSC conditioned medium (CM) and HUVEC medium (v/v 1:1) were seeded onto each well and incubated for 16 h. The morphology of tube‐like structure were observed and recorded by a bright‐field microscope. The total length of the branching tubes was quantified by ImageJ with an Angiogenesis Analyzer plugin (NIH). CM was collected from the same number of MSC (2 x 10^5^ cells/12‐well) seeded on culture plate for 16 h. In some experiments, recombinant HGF (1 ng/ml) was added to the CM for analyzing the stimulatory effect on tube formation.

### 4.6 Bone defect model and stem cell transplantation

All the animal procedures have been approved by Animal Experimentation Ethics Committee (AEEC) of CUHK. We created the segmental bone defect in the femur of adult SCID mice (12‐week old), which were purchased from the Laboratory Animal Services Centre (LASEC) and maintained at the Animal Holding Core of CUHK. (Wan *et al*, 2008). The mice were anaesthetized using Xylazine (5 mg/kg) and Ketamine (40 mg/kg) cocktail. The surgical procedure was performed in the right femur under sterile conditions. The middle portion of the of femur was exposed through an incision at the lateral side of the thigh. A 3 mm long segmental defect was created using a fine surgical saw. The defects were transplanted with the 3D Alginate‐Gelfoam complexes incorporated with iPSC‐derived MSC. Five mice were used in each group. Generation of the Alginate‐Gelfoam‐MSC complexes was adapted from an established protocol (Wang *et al*, 2016). The Gelfoam was trimmed into a cylindrical structure (2 mm in diameter and 3 mm in length), and was pre‐humidified with sterile water and dried in sterilized gauze. WS (WRN^−/−^) or C21 (WRN^+/+^) MSC were collected and resuspended in 4% alginate solution in 0.9% sodium chloride solution. The Alginate/cell mixture (1 × 10^6^ cells / 10 μl) was carefully dropped into the prepared Gelfoam scaffold followed by immersing with 102 mM calcium chloride solution. The 3D Alginate‐Gelfoam‐MSC complexes were formed in the calcium chloride solution for three minutes and then washed twice with 0.9% sodium chloride. The 3D complexes were maintained in the culture incubator at 37°C for 24 hr before transplantation. The bone defects were stabilized with an external fixator. The wound was closed with surgical sutures. At day 14 post‐surgery, the mice were sacrificed by cervical dislocation. The right femurs were harvested, fixed with 10% formalin, decalcified with 10% EDTA solution for 3 weeks at 4°C and further processed for paraffin embedding.

### 4.7 Cutaneous wound healing model and stem cell transplantation

All the animal procedures have been approved by Animal Experimentation Ethics Committee (AEEC) of CUHK. Genetically diabetic C57BLKS/Jlar‐+Lepr*^db^*/+Lepr*^db^* mice (db/db mice) were used in the wound healing model due to the slow wound healing rate (Greenhalgh *et al*, 1990). The mice were purchased from the Laboratory Animal Services Centre (LASEC) and maintained at the Animal Holding Core of CUHK. The back hair of the mice was firstly shaved and then the back skin was punched by a sterile puncher under anesthesia condition. The diameter of created wound is 4.5 mm. MSC were suspended in 50 μl matrigel solution and transplanted to the wound bed. Then the wounds were covered with Tegaderm films (3 M). The db/db mice were monitored every day after surgery. Six ‐ eight mice were included in each group. At the end of the experiment, mice were sacrificed and skin tissues surrounding the wounds were harvested for histological analyses.

### 4.8 Measurement of wound

Following MSC treatment, we measured the size of wounds on each mouse on day 0, 3, 6 and 9 using a ruler. Wound closure was calculated as change of the size of the wound at each time point relative to the size at day 0.

### 4.9 Histological analysis and immunohistochemistry

We harvested the full thickness skin tissues of wounded area 9 days after MSC treatment. Tissues were fixed overnight in 4% paraformaldehyde solution and embedded in paraffin with the dermis side faced down to the cassette. Tissues were sectioned and stained with Hematoxylin and Eosin (H&E) solutions. Sections with a clear boundary of the wound were chosen to show. For immunohistochemistry, cross‐sections of the skin tissues were harvested and processed as described above. Immunohistochemistry staining in the bone sections of the bone defect region or the full skin tissues was performed using standard protocols as recommended by the manufacturer (EnVision System; Dako). Briefly, the paraffin embedded sections were processed for antigen retrieval by digestion with proteinase K (20 µg/ml) for 10 min, and then incubated with primary antibody against Endomucin (Abcam, ab106100; dilution 1:100), CD31 (Abcam, 28364; dilution 1:50), VEGF (Abcam, ab1316; dilution 1:200) and α‐SMA (Abcam, ab23575; dilution 1:200) overnight at 4°C. A horseradish peroxidase (HRP) streptavidin detection system was used to detect the immunoactivity followed by counterstaining with hematoxylin (Sigma). The bone sections incubated with 1% nonimmune serum PBS solution served as negative controls. Quantitation of the Endomucin and CD31 positive vessel area in the bone defect region was performed using ImageJ software.

### 4.10 Masson's trichrome staining

Slides are deparaffinized and rehydrated through a series of alcohol (from 100% to 70%) and washed in water. Slides are first stained in Weigert's iron hematoxylin working solution for 15 minutes and washed in running water for 15 minutes. Then a second stain in Biebrich scarlet‐acid fuchsin solution for 15 minutes was performed. Slides were incubated in phosphomolybdic‐phosphotungstic acid solution until collagen was not red. Then slides were transferred to aniline blue solution and stained for 15 minutes. Finally, slides were washed in distilled water and differentiated in 1% acetic acid solution for 5 minutes. Hydrated slides were mounted with a coverslip. Collagen was stained as blue whereas cytoplasm, muscle and erythrocytes were stained as red.

### 4.11 Western blot and ELISA

For Western blot, SDS buffer was used for cell lysis. The whole‐cell protein was quantified by BCA assay kit (Thermo Fisher). The standard protocol of Western blot was performed as we previously described (Tu *et al*, 2018). The primary antibodies used in this study were listed as follows: PI3 K (Cell Signaling, 4252S, 1:1000), AKT (Cell Signaling, 9272, 1:1000), p‐AKT (Cell Signaling, 4060S, 1:2000), WRN (Sigma‐Aldrich, clone 195C, 1:500), GAPDH (Cell signaling, 5174S, 1:10000). After incubation with the appropriate secondary antibodies, signals were visualized by enhanced chemiluminescence (GE systems). For ELISA assay, 5 × 10^5^ MSC were seeded in 6‐well plate (triplicate) for 24 h. Supernatant was collected for the secreted HGF was measured by an ELSIA kit purchased from Abcam (ab100534), according to the manufacturer's instruction.

### 4.12 RNAI knockdown and pharmacological inhibition of WRN

We knocked down genes using shRNA cloned in pLVTHM lentivector (Addgene No. 12247). Pseudo‐lentivirus packaged in 293 T cells was used to infect MSC which were subsequently GFP‐sorted by FACS. We used siRNA (GenePharma) to knock down WRN in primary MSC using siIMPORTER (Millipore). To inhibit WRN, we treated cells with NSC19630 (Millipore) at 1‐3 μM for 24 h. DMSO was used as solvent control.

### 4.13 RNA‐SEQ and quantitative RT‐PCR

Total RNA from MSC was extracted by Trizol Reagent and purified in Direct‐zol RNA columns (Zymo Research). RNA was quantified using NanoDrop2000 spectrophotometer (Thermo Fisher). For RNA‐seq, whole transcriptome expression was performed by the NGS platform from GROKEN Bioscience (GROKEN), according to GROKEN’s suggested procedures for library construction and data analysis. Bioinformatics was also provided by GROKEN. The raw data was deposited Gene Expression Omnibus (GSE137856). For RT‐qPCR, mRNA was first converted to cDNA using PrimeScript RT‐PCR kit (Takara). Real‐time qPCR was performed using SYBR‐based method in QuantStudio 7 Flex system (Applied Biosystems). GAPDH was used as normalization control.

### 4.14 Statistics

Data are presented as means ±SD for three independent experiments. Statistical comparison in different experiments was calculated by GraphPad Prism 5 using two‐way ANOVA or Student's *t* test. *p* < 0.05 was considered statistically significant.

The author would like to apologize for the inconvenience caused.
