# Supplementary material for: Genetic correction of Werner syndrome gene reveals impaired pro‐angiogenic function and HGF insufficiency in mesenchymal stem cells
Source: Aging Cell. 2020 Apr 22;19(5):e13116. doi: 10.1111/acel.13116 (PMC7253065; doi:10.1111/acel.13116)
Supplement: Supplementary file 1 [file ACEL-19-e13116-s001.pdf]

Table S1. Karyotypes of iPSC clones derived from Werner syndrome AG00780

iWS780-1.2	46,XY,inv(2)(p23q35),del(4)(p16)
iWS780-1.5	46,XY,inv(2)(p23q35),del(4)(p16)
iWS780-1.9	46,XY,t(5;16)(q11.2;q22),t(8;9)(q24.1;q22),t(18;22)(p10;q10)
iWS780-2.1	46,XY,t(5;16)(q11.2;q22),t(8;9)(q24.1;q22),t(18;22)(p10;q10)
iWS780-2.7	46,XY,add(1)(q42)
iWS780-2.8	46,XY
iWS780-2.9	46,XY,inv(2)(p23q35),del(4)(p16)
iWS780-2.10	46,XY
iWS780-2.14	47,XY,+13

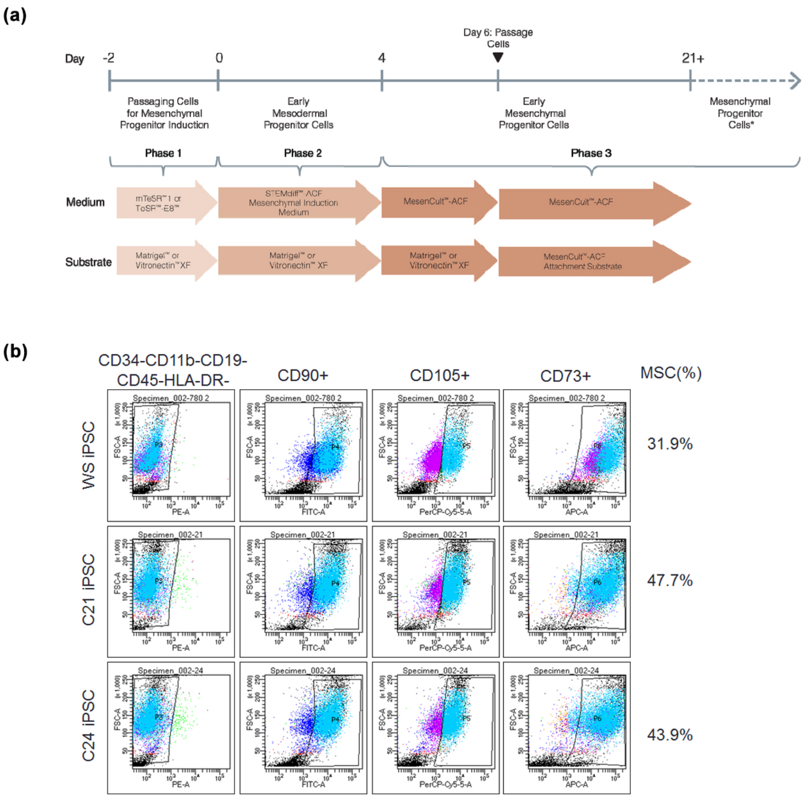

Supplementary Figure 1

(a) Schematic diagram showing the timeline and culture conditions for the differentiation of MSC from iPSC/ESC. Cells were sorted after 21 days of diferentiation. (b) Gatings of MSC-negative markers (CD34-/CD11b-/CD19-/CD45-/HLA-DR-) and positive markers (CD90+/CD105+/CD73+). Percentage of gated cells was shown as the efficiency of differentiation.

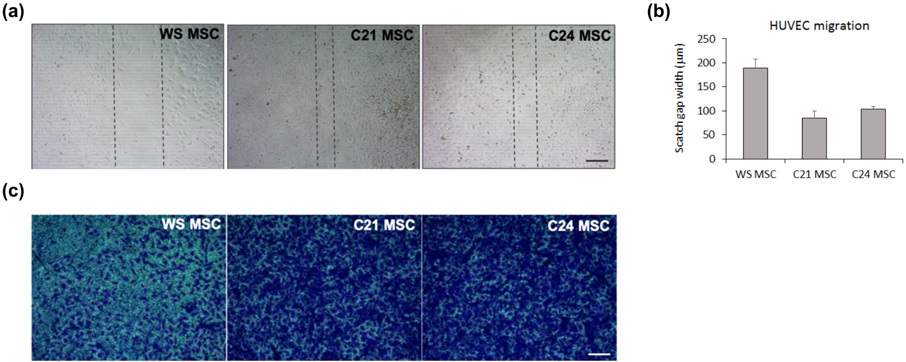

Supplementary Figure 2

(a) Cell-based wound healing scratch assay was used to examine the effect of MSC CM on promoting HUVEC migration. Phase contrast images were taken 16 hours after HUVEC stimulated with the CM. (b) Quantification of (a). Triplicate wells were analyzed and averaged. (c) Transwell-based cell invasion assay was used to examine the effect of MSC CM on promoting HUVEC invasion through a thin layer of matrigel. After 16 hours of seeding, cells outside the transwell were stained by Crystal violet and imaged.
Scale bar: 100 μm.

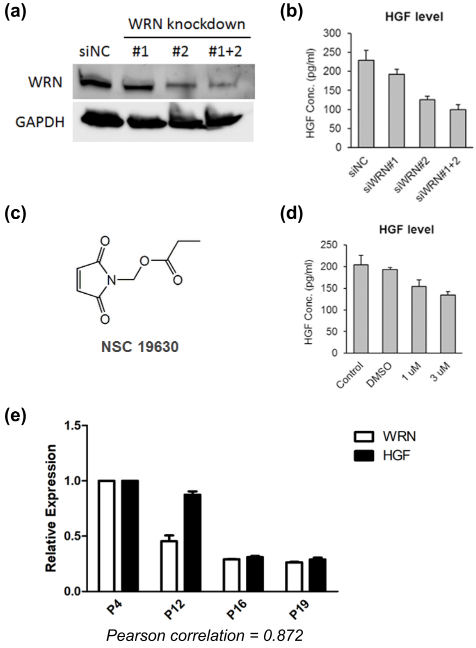

Supplementary Figure 3. Knockdown and pharmacological inhibition of WRN in primary MSC resulted in decreased HGF expression.
(a) Knockdown of WRN by siRNA. Western blot was performed after 48h of transfection. (b) HGF level was measured by ELISA after WRN knockdown. (c) NSC19630 is a specific inhibitor for WRN helicase. (d) MSC was treated with 1-3 μM NSC19630 for 24h. HGF level was measured by ELISA. Correlation between WRN and HGF expression levels was calculated by the expression levels of the two genes at different passages.

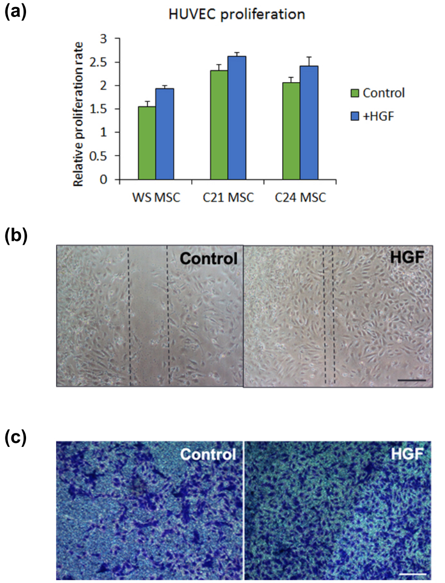

Supplementary Figure 4. Effect of exogenous HGF on the proliferation, migration and invasion of HUVEC. Methods of the assays were similar to Supplementary Figure 2.

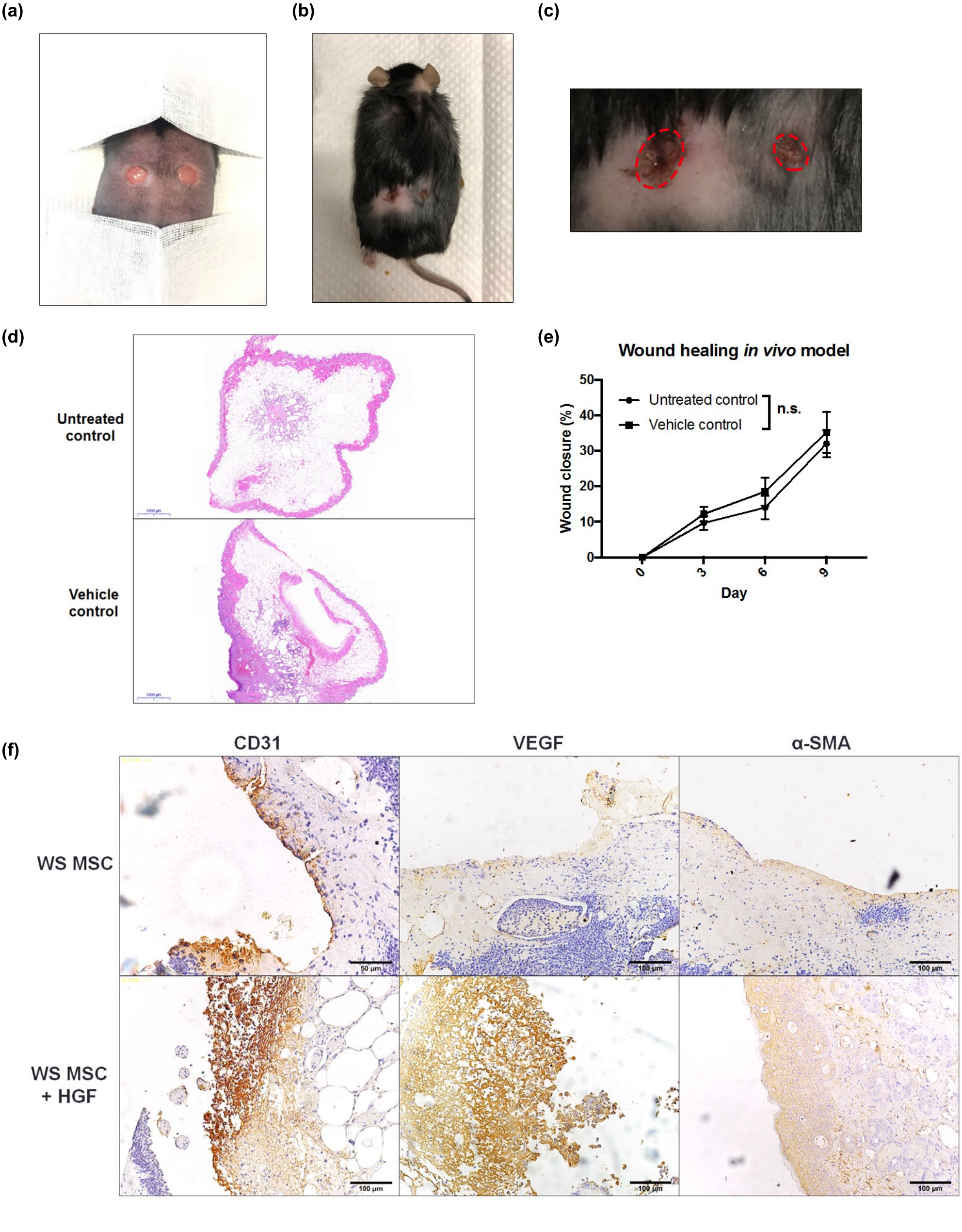

Supplementary Figure 5

(a) Generation of incisional wounds on the back of diabetic mouse before MSC treatment. (b) Wounds after 9 days of MSC treatment. (c) Magnified view of the wounds in (b). Wound areas were indicated circles. Left wound was treated with C21 MSC, whereas right wound was treated with WS MSC. (d) Gross H&E staining of the wounds with no treatment (untreated control) and vehicle (matrigel) control. (e) Wound healing process comparing untreated and vehicle controls. (f) Immunohistochemistry analysis of CD31, VEGF and α-SMA with the wounds treated with WS MSC or WS MSC with HGF protein.
Scale bar: 1000 μm in (d), 100 μm in (f); n.s. not significant.

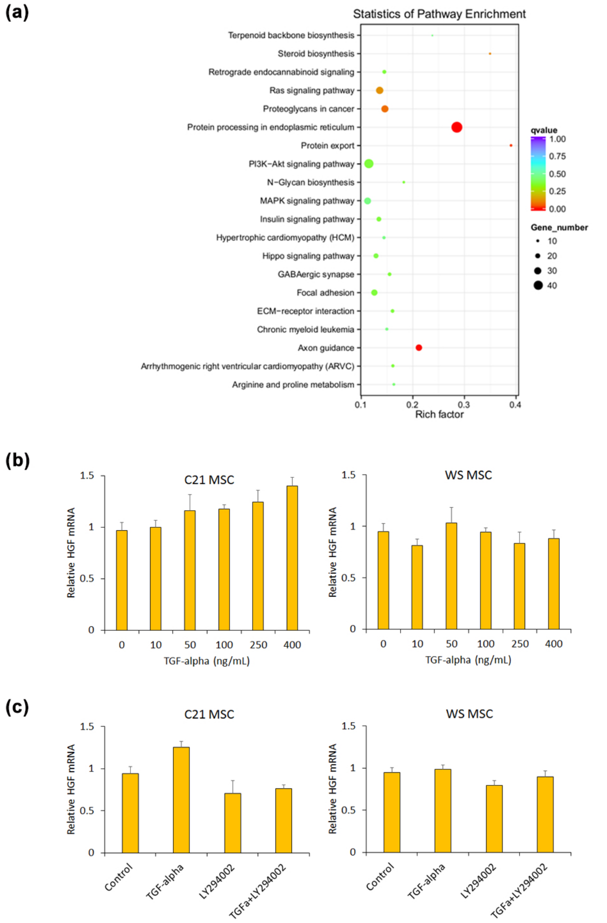

Supplementary Figure 6

(a) Pathways altered in WS MSC.The KEGG Enrichment Scatter Plot showed the most significantly enriched 20 pathways in C21/C24 MSC compared to WS MSC. (b) Expression of HGF in response to TGF-alpha treatment. MSC were treated with various concentrations of recombinant TGF-alpha protein for 24h in serum-free medium. (c) Expression of HGF in response to TGF-alpha, LY294002 or both. Relative mRNA level was examined by qPCR.

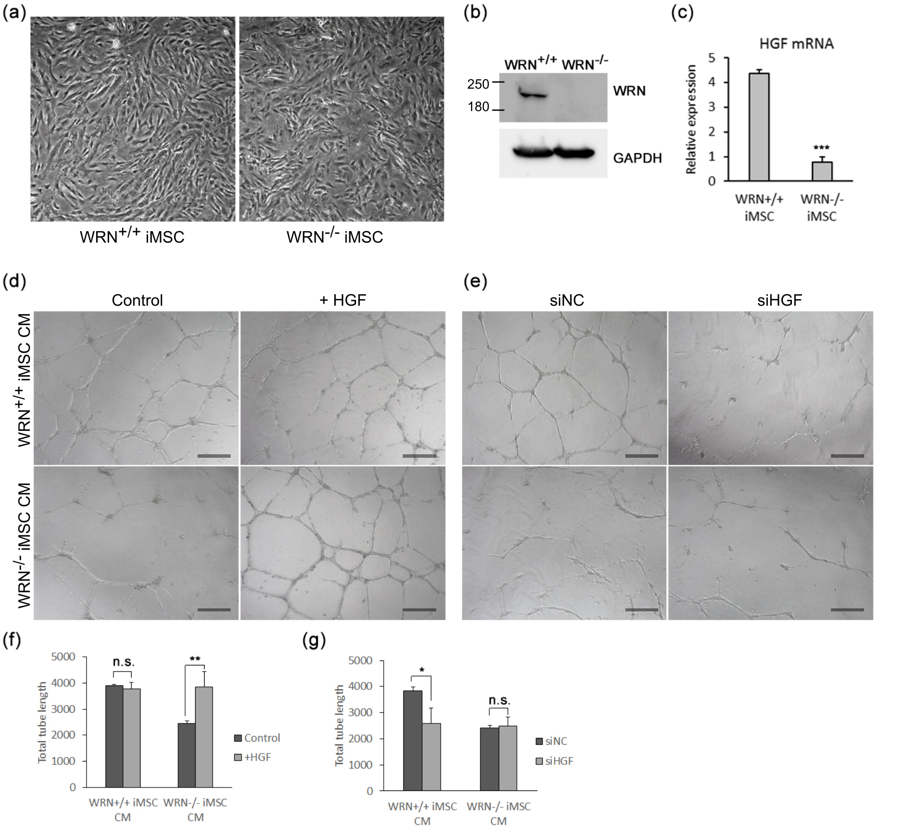

Supplementary Figure 7. Reduced HGF expression and impaired pro-angiogenesis in ESC-dervied MSC model of WS.

(a) $WRN^{+/+}$ and $WRN^{-/-}$ MSC were differentiated from wild-type and WRN-mutant H1 ESC. WRN-mutant line was generated by CRISPR/Cas9. (b) Expression of WRN protein in MSC by Western blot analysis. (c) Expression of HGF in MSC. (d, f)) Tube formation assay showed differential pro-angiogenic activity using HUVEC exposed to conditioned medium (CM) collected from $WRN^{+/+}$ and $WRN^{-/-}$ MSC. Addition of HGF to $WRN^{-/-}$ CM enhanced angiogenesis. (e, g) Knockdown of HGF in $WRN^{+/+}$ MSC resulted in reduced angiogenesis of HUVEC.
Scale bar: 200 μm; .**P* < 0.05; ***P* < 0.01; ****P* < 0.001; n.s. not significant.
